# Functional quantification of oral motor cortex at rest and during tasks using activity phase ratio: A zero-setting vector functional near-infrared spectroscopy study

**DOI:** 10.3389/fphys.2022.833871

**Published:** 2022-09-23

**Authors:** Masaaki Arai, Hikaru Kato, Toshinori Kato

**Affiliations:** ^1^ Department of Oral Biomedical Research, Total Health Advisers Co., Chiba, Japan; ^2^ Department of Brain Environmental Research, KatoBrain Co., Ltd., Tokyo, Japan

**Keywords:** oral frailty, initial dip, activity phase ratio, cerebral oxygen metabolism, dental medicine, fNIRS, functional near-infrared spectroscopy, oral motor cortex

## Abstract

Oral frailty associated with oral hypokinesia may cause dementia. Functional near-infrared spectroscopy (fNIRS) can be used while the participants are in seating position with few restrictions. Thus, it is useful for assessing brain function, particularly oral motor activity. However, methods for identifying oral motor cortex (OMC) activation *via* the scalp have not been established. The current study aimed to detect OMC activation, an indicator of activity phase ratio (APR), which reflects increased oxygen consumption (0 < [deoxyhemoglobin (*ΔDeoxyHb*) or 0 < {[*ΔDeoxyHb*- oxyhemoglobin (*ΔOxyHb*)/√2]}, *via* fNIRS to accurately identify local brain activity. The APR, calculated *via* zero-set vector analysis, is a novel index for quantifying brain function both temporally and spatially at rest and during tasks. In total, 14 healthy participants performed bite tasks for 3 s per side for 10 times while in the sitting position. Then, time-series data on concentration changes in *ΔOxyHb* and *ΔDeoxyHb* were obtained *via* fNIRS. The anatomical location of the OMC was determined using a pooled data set of three-dimensional magnetic resonance images collected in advance from 40 healthy adults. In the zero-set vector analysis, the average change in *ΔOxyHb* and *ΔDeoxyHb* concentrations was utilized to calculate the APR percentage in 140 trials. The significant regions (z-score of ≥2.0) of the APR and ΔOxyHb in the task were compared. During the bite task, the APR significantly increased within the estimated OMC region (56–84 mm lateral to Cz and 4–20 mm anterior to Cz) in both the right and left hemispheres. By contrast, the *ΔOxyHb* concentrations increased on the bite side alone beyond the OMC region. The mean APR at rest for 2 s before the task showed 59.5%–62.2% in the left and right OMCs. The average APR for 3 s during the task showed 75.3% for the left OMC and 75.7% for the right OMC during the left bite task, and 65.9% for the left OMC and 80.9% for the right OMC during the right bite task. Interestingly, the average increase in APR for the left and right OMCs for the left bite task and the right bite task was 13.9% and 13.7%, respectively, showing almost a close match. The time course of the APR was more limited to the bite task segment than that of *ΔOxyHb* or *ΔDexyHb* concentration, and it increased in the OMC. Hence, the APR can quantitatively monitor both the resting and active states of the OMC in the left and right hemispheres. Using the zero-set vector-based fNIRS, the APR can be a valid indicator of oral motor function and bite force.

## Introduction

Oral frailty should be prevented to maintain brain health (Horibe et al., 2018). Recent studies have shown that cognitive function decline and oral motor function decline are correlated with each other (Kugimiya et al., 2019; Takeuchi et al., 2017). The oral motor cortex (OMC), which is associated with oral movement, was first investigated *via* positron emission tomography (PET) (Fox et al., 2001). Subsequently, brain activation during gum chewing (Onozuka et al., 2002), clenching (Tamura et al., 2002), and mastication (Matsushima et al., 2005) was assessed *via* functional magnetic resonance imaging (fMRI). Thus, it is extremely important to understand the association between oral exercise and brain function from the perspective of dementia prevention.

Brain activity during rest differs between the supine and sitting positions (Thibault et al., 2014). In addition, gravity-induced mandibular retracted position may occur in the supine position. Prefrontal activation varies between the mandibular retracted and normal mandibular positions (Otsuka et al., 2015). However, patients who undergo PET or fMRI for the assessment of brain activity, including the OMC, should be in the supine position.

Functional near-infrared spectroscopy (fNIRS) has few limitations in terms of posture and movement during measurement (Kato et al., 1993; Kato, 2018). Therefore, fNIRS can be an optimal technique for assessing brain function during daily activities performed in the sitting or standing position (Kasahara et al., 2007; Kasahara et al., 2008). Brain activity during swallowing (Kober et al., 2015), oral care (Fujii et al., 2015), and clenching (Shibusawa et al., 2009) was evaluated *via* fNIRS. However, brain responses localized in the primary motor cortex (M1) were not detected. Clenching is an involuntary, forceful biting of the upper and lower teeth. Several aspects of this mechanism, such as bite duration, are unknown. By contrast, first bite is an oral movement that lasts only for a few seconds, and it plays an important role in the process of mastication (Dan et al., 2007). fNIRS methods for measuring OMC activity for a few seconds during bite have not been established yet.

Thus, fMRI and fNIRS have been used to evaluate brain function, particularly oral motor activity. Both modalities have a common method of detecting brain response to a task by comparing the resting state with a certain task. However, challenges in the quantitative assessment of the resting state of either modality remains unresolved.

Reports of many fNIRS studies have used increases in oxyhemoglobin concentration (OxyHb) and decreases in deoxyhemoglobin concentration (DeoxyHb) as brain activity. However, these are indicators of cerebral oxygen supply, and there is a possibility that cerebral blood supply also occurs around the area where the brain activity occurred (Kato, 2004). In addition, it has been pointed out that distinguishing between cutaneous blood flow and motion artifact contamination from the cortical activity is difficult when using OxyHb as the sole indicator of measurement (Kirilina et al., 2012; Kirilina et al., 2012; Miyazawa et al., 2013). Therefore, in order to identify brain activity more accurately, it is necessary to detect the initial dip caused by a decrease in OxyHb and an increase in DeoxyHb as an indicator of cerebral oxygen consumption and to use the indicator from the vector-based fNIRS, which simultaneously uses DeoxyHb and OxyHb (Kato, 2022).

The vector-based fNIRS has been applied for early dip detection and brain–computer interface studies (Hong and Naseer, 2016; Kato, 2013; Kato, 2018; Yoshino and Kato, 2012). Recently, it has played a role in the identification of oxygen metabolism during exercise training (Kato, 2022). Zero-set vector-based fNIRS has been utilized to quantitatively monitor the resting state of individual brain measurement channels (Kato, 2021).

We hypothesized that OMC function in biting movements such as eating can be validated using the activity phase ratio (APR), a novel index calculated *via* vector-based fNIRS. The current study aimed to quantitatively monitor OMC activity in the resting state and during a 3-s bite period *via* zero-set vector-based fNIRS using APR.

## Methods

### Participants

In total, 14 healthy adults who were right-handed based on the Edinburgh Handedness Inventory [mean age: 20.1 years, standard deviation (SD): 0.3 years; 11 men, 3 women] were included in the analysis. The experimental procedure was performed in accordance with the principles of the Declaration of Helsinki. Furthermore, this research was approved by the ethics committee of KatoBrain Co., Ltd. All participants received full explanation of the procedures and provided a written informed consent for study participation.

### Determination of chewing side preferences

Chewing side preferences (CSPs) were defined as the initial chewing side of the participants (Ozaki, 2002). A cotton roll (1.0 cm × 0.8 cm) was placed in the center of the tongue. Then, the initial chewing side was observed and recorded by a dentist. The side used for ≥2 times in three trials was considered as the CSP. In total, 10 participants (71.4%; seven men, three women) had right CSP, and four participants (28.6%; all men) had left CSP. The percentage of participants with right CSP was consistent with that in previous reports (Ozaki, 2002; Zamanlu et al., 2012).

### Measurement areas

**FIGURE 1:**
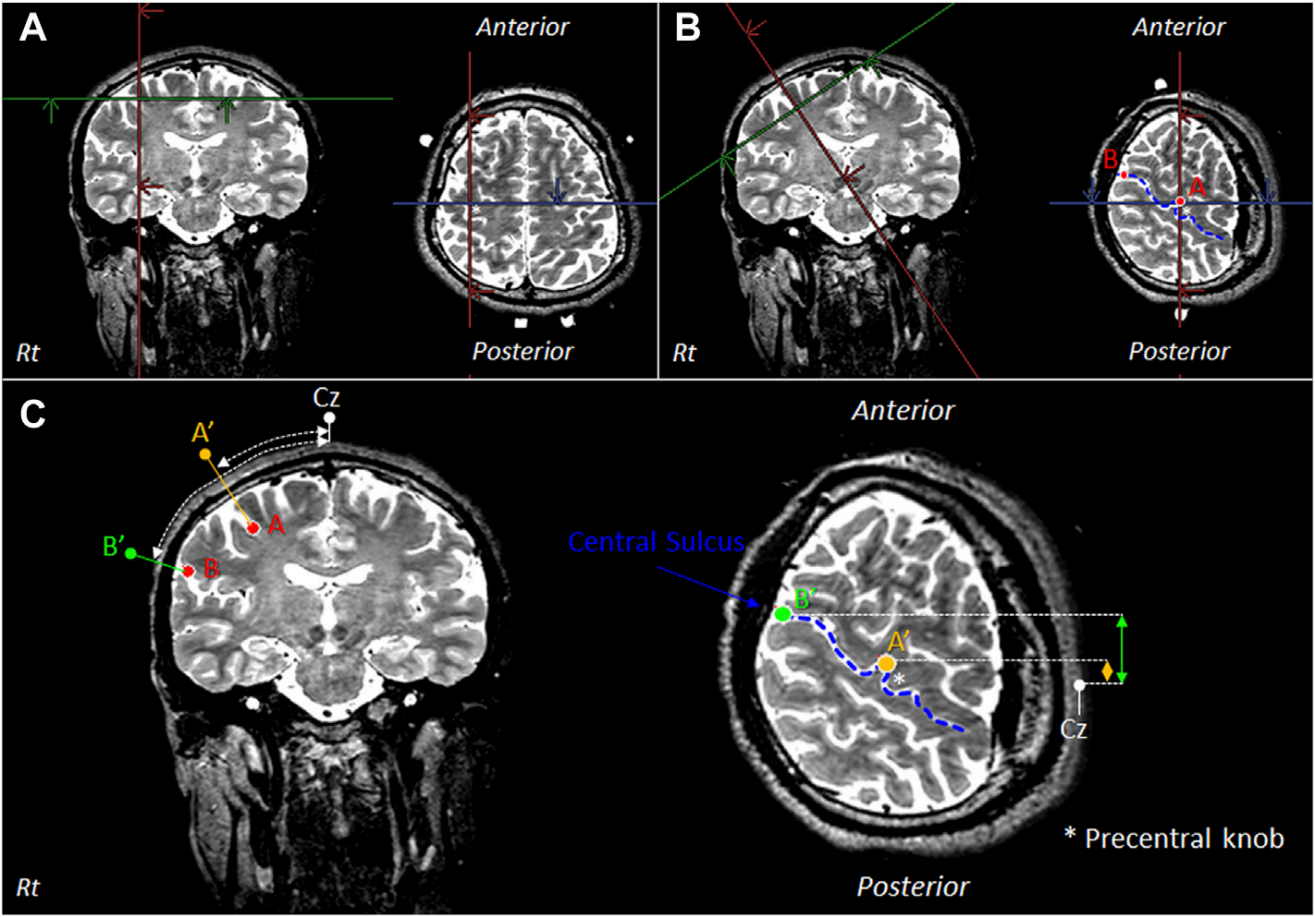
MRI for selecting the OMC measurement region. **(A)** Extract the outer edge of the precentral knob in the primary motor cortex (M1) and define it as point A. **(B)** Display an image in which point A is tilted as an axis perpendicular to the skull. In the displayed image, the outer edge of the central sulcus is defined as point B. The blue line indicates the central sulcus of the right hemisphere. **(C)** Points A′ and B′ are defined as points passing through points A and B and perpendicular to the skull. There is OMC in the area between points A′ and B′. Thus, the distances between Cz, point A′, and point B′ in the longitudinal direction and lateral direction were measured. OMC: oral motor cortex. Cz: location of vertex on the scalp. Rt: right.

OMC identification was performed *via* pre-measured identification of the target cortical sites (Murakoshi and Kato, 2006), using distances between reference points on the scalp from the MRI images of 40 healthy adults [average age: 32.2 (SD: 5.8) years; 20 men and 20 women]. The OMC region was defined as the region of interest (ROI). The OMC measurement sites were established using three-dimensional (3D) T2-weighted data. The Achieva 3.0-T Quasar Dual MRI Scanner (Philips Co.) was used. The pulse sequence was as follows: spin-echo sequence–TE: 255 ms, TR: 2,700 ms, flip angle: 90°, matrix: 25 cm × 25 cm, and resolution: 1.0 × 1.0 × 1.0 mm^3^. For MRI data analysis, the AZEWIN DICOM viewer (AZE Co.) was utilized. Figure 1A,B,C show the MRI method used to select the OMC measurement region. The OMC is estimated to be located outside the precentral knob (Yousry et al., 1997), which is the region of hand movement. The blue line in the Figure 1B,C indicates the central sulcus of the right hemisphere.

**FIGURE 2:**
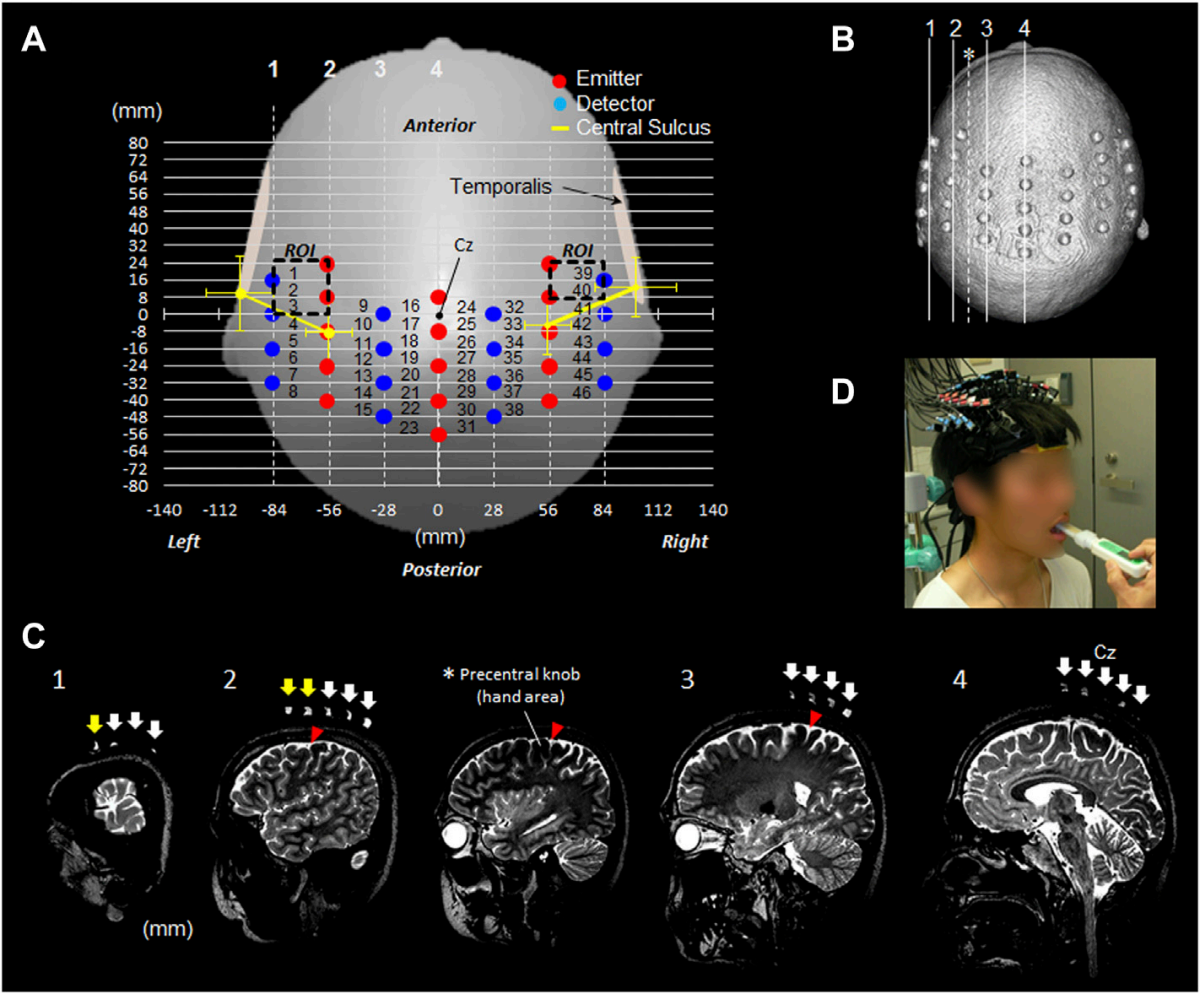
fNIRS measurement settings. **(A)** fNIRS 46-channel arrangement and the estimated bilateral OMC. **(B)** Head image reconstructed from 3D T2-weighted MRI taken with a registration marker, which indicates the probe position. The four lines from 1 to 4 correspond to the positions in the image in Figure **(C)**. **(C)** Sagittal images 1–4 along the registration marker are shown. The sagittal image presented with an asterisk indicates a slice passing through the precentral knob between rows 2 and 3. Among the arrows indicating the probe position, the yellow arrow of slices 1 and 2 indicates the position of the pair probe sandwiching the part corresponding to the OMC. **(D)** Participant wearing a probe during experimental tasks. OMC: oral motor cortex. ROI: region of interest. Cz: location of vertex on the scalp.

Figure 2A shows the position of the OMC measured to determine the location where the probe should be attached before starting the fNIRS experiment. The measurement ranges were 24 mm forward, 56 mm rearward, and 84 mm outside bilaterally from the Cz reference point. Cz is a landmark of the international 10–20 system for electroencephalogram electrode placement. The left and right OMCs were considered as the ROI surrounded by the dotted rectangle. The positions of the OMC on the scalp were 56–84 mm outside and 4–20 mm anterior based on Cz. The estimated right OMC was located at channels 39 and 40 and the estimated left OMC at channels 1, 2, and 3.

The attachment was mounted corresponding with Cz between the first and second emitter probes in the center. The red and blue circles indicated the emitter and detector, respectively. The distance between the emitter and detector was 3.0 cm. The distances between the channels were set to 2.8 cm in the horizontal direction and 0.8 cm in the sagittal direction, which ensured a sufficiently high resolution to secure cortical data (Kawaguchi et al., 2004). The yellow line in the Figure 2A indicated the estimated direction of the central sulcus outside the precentral knob corresponding to the region of voluntary hand movement (Figure 1A,B). With reference to Cz on the scalp, in the left hemisphere, the central sulcus runs outside the precentral knob in the direction connecting the points 8.6 ± 6.9 mm (mean ± SD) posterior to Cz and 56.0 ± 5.9 mm outside from the points 9.7 ± 8.7 mm anterior and 101.3 ± 8.5 mm outside Cz.

In the right hemisphere, the central sulcus runs outside the precentral knob in the direction connecting the points 5.0 ± 6.9 mm posterior to Cz and 55.9 ± 5.9 mm outside from the points 12.5 ± 6.8 mm anterior and 101.1 ± 9.0 mm outside Cz.

### fNIRS measurement

A multichannel fNIRS (FOIRE-3000; Shimadzu Corporation, Japan) with 15 irradiation probes and 16 detection probes was used. Furthermore, a self-made measurement probe with 46 channels was utilized (Figure 2A,D). Changes in oxyhemoglobin (ΔOxyHb) and deoxyhemoglobin (ΔDeoxyHb) concentrations were monitored by detecting scattered light with three wavelengths of near-infrared light (780, 805, and 830 nm) irradiated to the scalp. Conversion from absorbance to ΔOxyHb was performed inside the device using a method by Matcher et al., 1995. Measurement was performed in a continuous mode. The sampling time of ΔOxyHb was 85 ms. Event marks were recorded at the task start and end. To prevent temporal muscle artifacts, the measurement channel was mounted more inside the origin of the temporalis muscle.

To prevent artifacts in the temporalis muscle, the measurement channel was mounted medial to the origin of the temporalis muscle. In addition, to evaluate the presence of motion artifacts in the scalp and temporalis muscles, the participants were instructed to open and close their mouths and perform a trial bite on a bite force meter prior to the experiment. Results confirmed the absence of artifacts in the OxyHb and DeoxyHb measurement waveforms, with rapid fluctuations caused by movement during the bite task. However, the presence of slow artifacts persisted until after the task was not confirmed.

After the experiment, a registration marker was placed at the probe placement site in one participant, and T2-weighted images were taken with 3-T MRI to confirm the probe location (Figure 2B,C). The other 13 participants did not undergo MRI after the experiment because the probes were placed using a pre-measured identification technique at the target cortical sites with distances between reference points on the scalp *via* MRI in healthy adults (Murakoshi and Kato, 2006).

As shown in Figures 1, 2, the position of the OMC in 40 participants was less sensitive to temporal muscle movements.

### Experimental procedures

The experimental task was set as the maximum bite task for 3 s with consideration of the characteristics of M1 related to force output (Cheney and Fetz, 1980; Hoffman and Luschei, 1980). The participants bit the bite force meter (GM10; Nagano Keiki Co., Ltd., Japan) with maximum force for 3 s in accordance with the vocal cue (Figure 2D). In the experimental task, the dentist placed the bite force meter sensor at the contact point between the first molar and second premolar on the lower jaw. The maximum bite force for 3 s was recorded using a bite force meter.

The right bite force was 49.0 ± 15.0 kg, and the left bite force was 50.0 ± 15.7 kg. Hence, there was no significant difference between the two bite forces (*p* = 0.405; paired *t*-test).

The participants performed right and left bite each for 10 trials. After each trial, the bite force meter was pulled from the oral cavity and maintained at rest. The interval between each trial varied from 10 to 15 s. Approximately 5 s before the next trial, the bite force meter was again set in the oral cavity. Therefore, it did not include jaw movement during the task. The data of the left and right bite tasks were obtained for each of the 140 trials. The left and right bite tasks were performed randomly to eliminate the order effect.

### Preparation of zero-set vector for fNIRS data

Five preparatory processing steps were required to calculate *ΔOxyHb* and *ΔDeoxyHb* concentrations and APR. The first step was the smoothing of OxyHb and DeoxyHb time-series data *via* fNIRS. The raw OxyHb and DeoxyHb data were low-pass filtered at 0.1 Hz (first-order Butterworth filter).

Baseline normalization and motion correction were not performed as the preprocessing step in the analysis due to the risk of distorting the phase of oxygen exchange (Kato., 2022).

The second step was the calculation of *ΔCOE* and *ΔCBV* in each trial using *ΔOxyHb* and *ΔDeoxyHb* data with Eqs 1, 2:

$$\Delta COE=\frac{1}{\sqrt{2}}\left(\Delta DeoxyHb-\Delta OxyHb\right)$$ (1)

$$\Delta CBV=\frac{1}{\sqrt{2}}\left(\Delta DeoxyHb+\Delta OxyHb\right).$$ (2)

**FIGURE 3:**
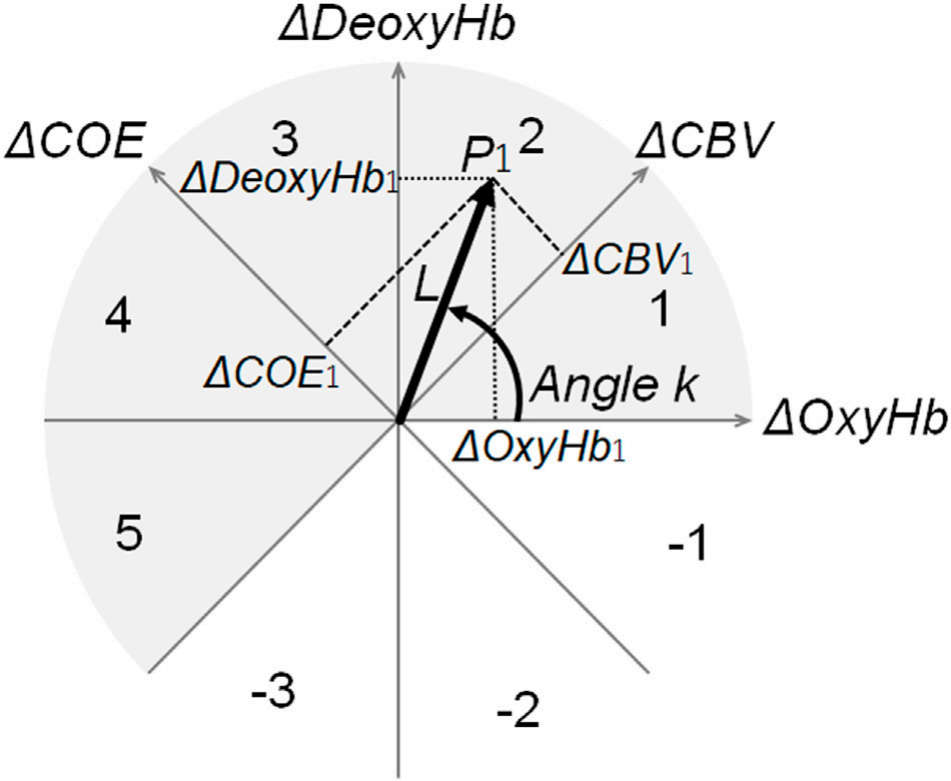
Polar coordinates for vector analysis. Vector polar coordinate plane used for vector analysis. By converting the vector connecting the origin and an arbitrary point P_1_ (*ΔOxyHb*_*1*_, *ΔDeoxyHb*_*1*_) to the *ΔCOE* axis and the *ΔCBV* axis, the coordinates of *ΔCOE*_1_ and *ΔCBV*_1_ are obtained. The number on the arc indicates the phase number. Among the eight quadrants divided by the four axes, the phase of the gray portion (*∆DeoxyHb* > 0 or *∆COE* > 0) implies low oxygenation or deoxygenation. Thus, these phases indicate increased brain activity (activation phase). By contrast, the phase of white (*ΔDeoxyHb* < 0 or *ΔCOE* < 0) showed minimal enhancement of brain activity (non-activation phase). *k* quantitatively indicates the phase of these oxygen metabolisms. *ΔOxyHb*: oxyhemoglobin. *ΔDeoxyHb*: deoxyhemoglobin. *ΔCOE*: cerebral oxygen exchange. *ΔCBV*: cerebral blood volume.

An orthogonal vector plane spanned by *ΔOxyHb* and *ΔDeoxyHb* (Kato, 2006; Kato, 2018) was used in the analysis of hemodynamic responses (Figure 3).

The third step was the offset of the start of each bite task in *ΔOxyHb* and *ΔDeoxyHb* to the vector origin to observe event-related responses.

The fourth step is the creation of a group of zero-set vectors of time-series changes in *ΔOxyHb* and *ΔDeoxyHb* concentrations at each given sampling time based on the two-dimensional diagram, as shown in Figure 3. This was defined as the zero-set vector.

The phase *k* and norm *L* values could be calculated using the zero-set vectors.

The fifth step was the calculation of all the phases (angles *k*) of the zero-set vector using Eq. 3.

The angle *k* between the zero-set vector and the positive *ΔOxyHb* axis could be calculated using Eq. 3.

The angle *k* between a vector and the positive *ΔOxyHb* axes could be calculated using Eq. 3:

$$k=\mathrm{Arctan}\left(\frac{\Delta DeoxyHb}{\Delta OxyHb}\right)=\mathrm{Arctan}\left(\frac{\Delta CBV}{\Delta COE}\right)+45{}^{\circ}\left(-135{}^{\circ}≦k≦225{}^{\circ}\right).$$ (3)

The oxygen exchange degree (*k* angle) was defined as a quantitative index of oxygen metabolism intensity based on the *ΔCOE-*to-*ΔCBV* ratio (or *ΔDeoxyHb*-to-*ΔOxyHb* ratio) obtained using Eqs 1, 2 (Kato, 2006; Kato, 2018).

The norm *L* between point P_1_ (*Δ* ${OxyHb}_{1}$ , *Δ* ${DeoxyHb}_{1}$ ) in Figure 3 and the origin can be described using the following equation:

$$L=\sqrt{{\left(∆OxyHb_{1}\right)}^{2}+{\left(∆DeoxyHb_{1}\right)}^{2}}=\frac{1}{\sqrt{2}}\sqrt{{\left(∆COE_{1}\right)}^{2}+{\left(∆CBV_{1}\right)}^{2}},$$ (4)

where *L* represents the intensity of Hb changes (OxyHb and DeoxyHb).

Each norm *L* value reflected the intensity of each zero-set vector, which is not analyzed in this study.

The phase *k* and norm *L* values could be calculated using the zero-set vectors. Each norm *L* value reflects the intensity of each zero-set vector, which was not analyzed in this study.

### Quantitative analysis of APR

After calculating the phases of the zero-set vector, the APR was calculated using Eq. 5.

As shown in Figure 3, phases 1–5 were defined as the activity phase. Therefore, the APR (%) was defined as the percentage of trials, with an activity phase out of the total number of trials. It was calculated for each measurement channel in each task.

The APR could be calculated using Eqs 5, 6, 7 (Kato, 2021):

$$APR\;\left(\%\right)=\frac{Number\;of\;trials\;shown\;in\;the\;activity\;phase\;at\;the\;sampling\;time\;\left(n\right)}{Total\;number\;of\;trials\;at\;the\;sampling\;time\;\left(n=140\right)}\times 100,$$ (5)

APR denominator=(number of participants) x (number of sites measured), (6)

APR numerator=number of activity phase trials among the total number of trials used as the denominator. (7)

This zero-set vector-based fNIRS study identified the areas where the task caused cortical activity in the ROI, since the resting state was quantified by the APR. The OMC was defined as the area of increased APR and oxygen metabolism in the combined force output task.

A measurement channel in which the z-score of the APR was ≥2.0 was considered a high ratio activity site. The OMC site was defined as the high ratio activity site in the bite force task.

In addition, we detected the measurement channels with z-scores of ≥2.0 for concentration changes in *ΔOxyHb*, which is a conventional activity index. Then, differences in the identifying sites of the OMC based on APR and *ΔOxyHb* concentrations were compared.

The cumulative sum of the four vector components (*ΔOxyHb*, *ΔDeoxyHb*, *ΔCOE*, and *ΔCBV*) for 3 s during the task was used for APR and *ΔOxyHb* mapping.

### Time trends in APR and *ΔOxyHb* and *ΔDeoxyHb* concentrations in the OMC

The APR was calculated every 0.51 s of sampling time to plot the time course of synchronicity between the increase in APR, and the task time was evaluated from the time course series for APR changes in the OMC. The *k* angle was calculated using the cumulative sum of *ΔCOE* and *ΔCBV* every 0.51 s, and the time-series change in the APR every 0.51 s was plotted. Using the APRs of the left and right bite tasks of each participant as paired data, differences in the APR between tasks were examined (paired *t*-test). Similarly, the waveforms of *ΔOxyHb* and *ΔDeoxyHb* plotted every 0.51 s were detected. A *p*-value of 0.05 was considered statistically significant.

## Result

### Quantitative mapping using the APR for identifying OMC location

**FIGURE 4:**
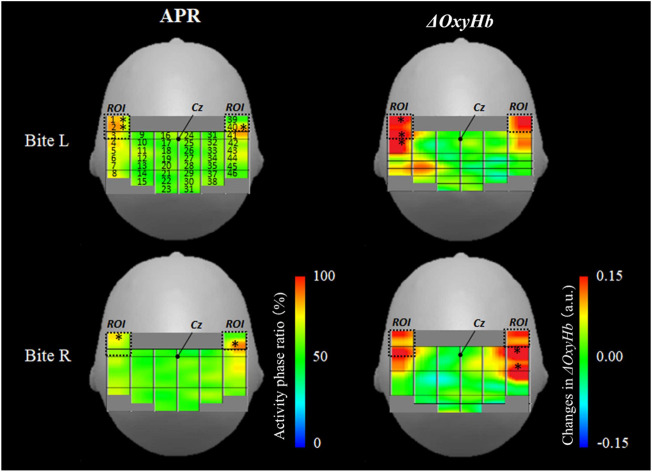
Activation map during unilateral bite task. The left column shows APR mapping in the right and left biting tasks. The right column shows *ΔOxyHb* mapping in the right and left biting tasks. An asterisk indicates a high ratio active site (z-score of >2.0). APR: activity phase ratio. L: left. R: right. *ΔOxyHb*: oxyhemoglobin.

Figure 4 shows mapping using the average value of the APR (%) and *ΔOxyHb* during the right and left biting tasks. In the analysis using the APR, channels 1, 2, and 40 had statistically significant results (z-score of >2.0) in the left bite task, and channels 1 and 40 had statistically significant results (z-score of >2.0) in the right bite task. Channels 1 and 40 were within the OMC area assessed *via* MRI. The area between these probes was located above the precentral gyrus and outside the area of the precentral knob, as shown in Figures 2C1,C2. These measurement channels were located in front of the outermost row of measurement probes, and these two channels had bilaterally symmetrical positions merely displaced by 8 mm in the anterior–posterior direction. Based on these results, the APR significantly increased only in the ROI channel corresponding to the OMC on both sides with respect to one-side bite.

In the analysis using *ΔOxyHb*, channels 1, 3, and 4 had statistically significant results in the left bite task, and channels 41 and 43 had statistically significant results in the right bite task (z-score of >2.0). The *ΔOxyHb* concentration significantly increased during the one-sided bite task only on the bite side and not on the opposite side. Hence, the *ΔOxyHb* concentration outside the OMC region increased on MRI. In the left biting task, the area in front of the central sulcus also increased. However, the most evident increase was detected behind the central sulcus (left bite: ch1 z-score = 2.8, ch3 z-score = 2.5, and ch4 z-score = 2.1; right bite: ch41 z-score = 3.0, ch43 z-score = 2.5).

### Quantitative detection of changes in the OMC at rest and during tasks using APR

**FIGURE 5:**
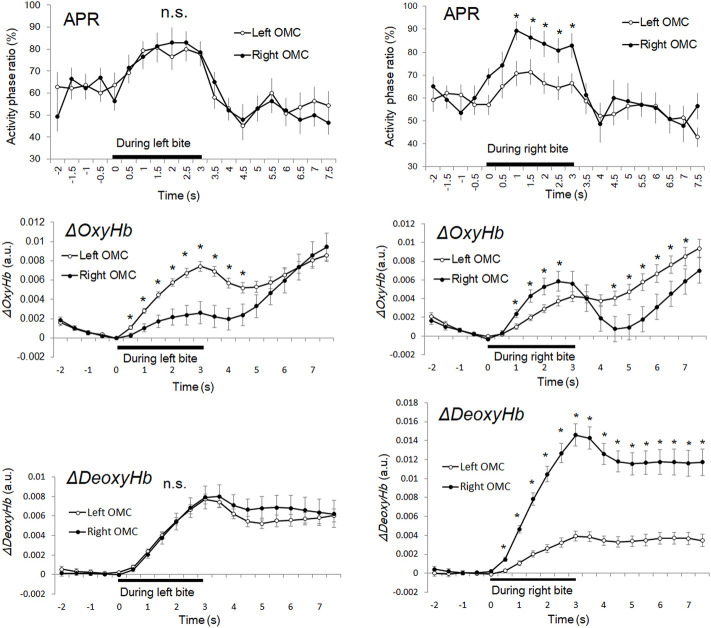
Time course series of APR, *ΔOxyHb*, and *ΔDeoxyHb* during unilateral bite task. The left column shows the time courses of the average APR of the right and left OMCs, respectively. The bar shows the standard error of the mean. An asterisk indicates a high signal site (z-score of >2.0) in which there was a significant difference between the right and left OMC. APR: activity phase ratio. APR: activity phase ratio. *ΔOxyHb*: oxyhemoglobin. *ΔDeoxyHb*: deoxyhemoglobin. OMC: oral motor cortex.

Figure 5 shows the time-series changes in APR and *ΔOxyHb* and *ΔDeoxyHb* concentrations in the left and right OMCs (left OMC: channels 1 and 2 and right OMC: channel 40). The APR on the bilateral OMC increased only during the 3-s task period for both right and left bite tasks. Meanwhile, the *ΔOxyHb* and *ΔDeoxyHb* concentrations increased after the task. The average APR for 2 s before the task, which can quantitatively indicate the level of oxygen metabolism in the OMC during the resting state, was 62.2% in the left OMC and 61.2% in the right OMC during the left bite task, and 60.0% in the left OMC and 59.5% in the right OMC during the right bite task.

The average APR for 3 s during the task showed 75.3% for the left OMC and 75.7% for the right OMC during the left bite task, 65.9% for the left OMC and 80.9% for the right OMC during the right bite task. Therefore, the average APR during the task increased by 13.2% in the left OMC and 14.5% in the right OMC of the left bite task and by 5.9% in the left OMC and 21.2% in the right OMC of the right bite task, compared with that before the tasks. This means that the percentage increase in oxygen consumption was about 1.1 times greater in the right OMC than in the left OMC for the left bite task and about 3.6 times greater for the right bite task. Thus, the difference in left and right OMC activities, which was scarcely observed during the left bite task, became more significant during the right bite task. Interestingly, the average increase in APR for the left and right OMCs for the left bite task and the right bite task was 13.9% and 13.7%, respectively, showing almost a close match.

In the left bite task, there was no significant difference between the APRs in the left and right OMCs in any time zone (*p* > 0.05). In the right bite task, the APR was significantly higher on average 16.7% in the right OMC than in the left OMC within 1.02–3.06 s (2.500 < *t* (13) < 3.553, *p* < 0.05).

The maximum peak APR values in the left bite task were 82.9% (z-score = 2.2, n = 140) at 2 s after task onset in the right OMC and 80.7% (z-score = 2.0, *n* = 140) in the left OMC, 1.5 s after task onset.

The maximum peak APR values in the right bite task were 89.3% (z-score = 3.2, *n* = 140) at 1 s after task onset in the right OMC and 71.4% (z-score = 2.0, *n* = 140) in the left OMC, 1.5 s after task onset.

Average APR for 4.5 s after the task showed 53.7% for the left OMC and 52.3% for the right OMC in the left bite task, 53.2% for the left OMC and 55.4% for the right OMC in the right bite task. Thus, compared to the pre-task, the average APR after the task showed a decrease of 8.4% in the left OMC and 8.9% in the right OMC for the left bite task, and a decrease of 6.8% in the left OMC and 4.3% in the right OMC for the right bite task, respectively. This means that the inactive phase, reflecting the oxygen supply, increased in the left and right OMCs after the task and remained in a state that did not return to the state before the task. Therefore, the peak APR value can be an indicator for detecting activities during short tasks within 3 s.

### Elevated *ΔOxyHb* and *ΔDeoxyHb* concentrations sustained after bite task

In both left and right bite tasks, *ΔOxyHb* showed a bimodal waveform and was increased at the beginning of the task and 1–2 s after the task (Figure 5). In the left bite task, the *ΔOxyHb* concentration of the left OMC was significantly higher than that of the right OMC (0.51–4.06 s; *p* < 0.001). The average ΔOxyHb concentration change in the left OMC during the task was approximately 2.7 times greater than the value in the right OMC. In the right biting task, the *ΔOxyHb* concentration of the right OMC was significantly higher than that of the left OMC (4.49–7.14 s and 1.02–2.55 s; *p* = 0.000–0.034). Thus, *ΔOxyHb* occurred strongly on the same side as the bite side, both in the left and right bite tasks.

The *ΔDeoxyHb* concentration of the bilateral OMC increased steadily from the beginning to the end of the right and left bite tasks. Subsequently, it decreased slightly, not returning to the starting point, but showing flattening with an increase. In the left bite task, there was no significant difference in *ΔDeoxyHb* concentrations between the left and right OMCs at any time point (*p* > 0.05), as in the APR time-series data. In the right bite task, the *ΔDeoxyHb* concentration of the right OMC was significantly higher than that of the left OMC (0.51–7.65 s; *p* < 0.001). The average *ΔDeoxyHb* concentration change in the right OMC during the task was approximately 4.4 times greater than the value in the left OMC.

Time-series data of both ΔOxyHb and ΔDeoxyHb concentrations increased during the task but showed divergence in post-task trends. In addition, the time-series data for ΔOxyHb and ΔDeoxyHb concentrations showed a rather different trend from the APR data for both right and left bite tasks.

## Discussion

### Identification of OMC using APR and MRI

This study showed that left and right OMC activation occurs even during unilateral bite. Based on previous studies on human mandibular movements, bilateral M1 activity is enhanced in oral motor tasks that do not distinguish between left and right bites. However, although unilateral dominance of movement increases bilateral OMC activity was unclear. The active sites of OMCs obtained from the scalp ranged from 56 to 84 mm laterally and from 4 to 20 mm anteriorly with respect to Cz. It showed an increase in APR during unilateral bite, which is consistent with the location of OMC assessed *via* MRI in 40 participants.

Since the APR activity in the OMC, indicating oxygen metabolism, occurs in the left and right hemispheres, bite movements are not significantly inhibited unless the bilateral OMCs are damaged. In a study comparing force production on the paralyzed and healthy sides (Kemppainen et al., 1999), it was difficult to recover the loss of finger palmar grip strength on the paralyzed side. However, there was no difference in maximum bite force between the left and right sides. The results of this study were consistent with those of previous ones. Closure muscles (e.g., the masseter, medial pterygoid muscle, and temporal muscle) are involved in biting and controlled by the trigeminal nerve motor branch from the OMC *via* the corticopontine tract. In the upper part of the trigeminal nucleus, the closure muscles are dually controlled by the bilateral cerebral hemispheres (Berkovitz, 2005). The results of this study are consistent with those of previous anatomical ones.

By contrast, ΔOxyHb, which is widely used in fNIRS analysis, had significant responses to not only the activity of the OMC itself but also the activity around the OMC. Meanwhile, the responses of APR and *ΔOxyHb* occurred 8 mm (1 channel width) apart. As shown in Table 1, previous functional brain studies were not able to individually detect the identified OMCs and surrounding brain activity.

**TABLE 1:** Functional brain studies of human mandibular movement *via* PET, fMRI, and fNIRS.

Author	Device	Index	Task	Task side	Performance data	M1^a^ activation
Fox et al. 2001	PET	rCBF (H_2_^15^O)	Speaking and reading aloud^b^	Bilateral	None	Bilateral
Onozuka et al. 2002	fMRI	BOLD	Gum chewing	Bilateral	None	Bilateral
Matsushima et al. 2005	fMRI	BOLD	Mastication (1 Hz)	Left or right	None	Left or bilateral
Shibusawa et al. 2009	fNIRS	OxyHb	Clenching	Right	% MVC^c^	Left (right is not measured)
Iida et al. 2012	fNIRS fMRI	OxyHb	Teeth tapping (1 Hz)	Bilateral	EMG^d^ activity	Bilateral
		BOLD				
Kanayama et al. 2015	fMRI	BOLD	Clenching	Bilateral	None	Bilateral
Arai et al. (present study)	Vector-based fNIRS	APR^e^	Object biting	Left or right	Bite force strength	Bilateral
		*ΔOxyHb*				
		*ΔDeoxyHb*				

a Primary moto area (M1).

b This was not a mandibular movement task study. However, it was the first study that identified OMC spatially using functional activation.

c Percent maximum voluntary contraction (MVC).

d Electromyography (EMG).

e Activity phase ratio (APR) (%).

With the use of phase *k*, not only the areas of increased oxygen consumption in brain activity but also the surrounding oxygen supplying regions can be detected. However, they are considered indistinguishable if BOLD or ΔOxyHb is used as an indicator (Kato, 2022).

### Temporal advantage of APR using zero-set vector-based fNIRS

The APR had different time-series changes compared with *ΔOxyHb* and *ΔDeoxyHb* concentrations and could quantify brain activity during oral movements. The APR trend could not be predicted *via* gross observation based on the trend of *ΔOxyHb* and *ΔDeoxyHb* time-series data. The APR was about 1.1 times higher in the right OMC than in the left OMC for the left bite task and about 3.6 times higher in the right bite task. Interestingly, the average increase in APR for the left and right OMCs for the left bite task and the right bite task was 13.9% and 13.7%, respectively, showing almost a close match. Since the participants did not show significant differences in the left and right bite strength, this suggests that in the absence of differences in bite strength, it is possible that bite muscles are controlled by the sum of activation of the left and right OMCs. Moreover, since all participants were right-handed, the APR results could be different for left-handed participants or children with deciduous teeth.

The APR recovered to about 60% of its pre-task value 1 s after the task. On the other hand, once the task is completed, neural activity is also expected to decrease, and as a result, ΔOxyHb and ΔDeoxyHb would be expected to return to their pre-task values. However, ΔOxyHb and ΔDeoxyHb increased after the task and did not return to their pre-task values. Thus, the use of ΔOxyHb and ΔDeoxyHb alone as indicators of neural activity may be misleading, as if neural activity is ongoing. The re-elevation of *ΔOxyHb* after the task may indicate cerebral blood supply to the OMC. However, the reason why the post-task *ΔDeoxyHb* did not return to pre-task values remains unclear.

An increase in APR indicates an elevated frequency of the initial dip, which represents increased oxygen consumption. The initial dip is more spatially limited than that associated with increased *ΔOxyHb* or blood volume changes (Ances et al., 2001; Kato, 2004; Kato, 2018; Suh et al., 2006). In fact, the initial dip is a spatially selective response in hand motor tasks (Akiyama et al., 2006). A brief 4-s increase in oxygen metabolism can be detected *via* phase evaluations using vector analysis (Kato, 2018). Values of 60%–70% at rest reflect the total percentage of phases -1, -2, and -3 in Figure 3, which is 30%–40%. Kato (2021) showed that the total APR of activity phases 1–5 was 65.7%, and the total percentage of inactivity phases -1, -2, and -3 was 34.3%, based on the APR measured from the right frontal region during 5 min of closed-eye rest.

In this study, the mean APR values (%) from the zero-set vector at rest exhibited different phase variations, with approximately 60% in the active phase and 40% in the inactive phase. In a previous study, the time course of the mean APR value (%) was examined in the Broca’s area of the left frontal lobe for approximately 4 s before subjects heard and spoke the word “lion” and thereafter. The results showed that the APR was about 60% in the 2 s before the task started, reached a maximum of 90% at 2 s after the task started, decreased to 30% at 5 s, and returned to 50% at 6 s (Kato, 2021). Thus, the reproducibility of the results, even if the measurement site and task differ, confirms that APR values are a novel quantitative measure of brain activity, not just in the resting state.

### Oral motor function in the sitting position

In the present study, the mean resting APR decreased by about 7% from about 60% before and after the bite task. It would be very interesting to see how the resting APR changes in daily life. Furthermore, the APR, which quantitatively indicates the state of brain activity at rest, may be a useful indicator of left and right OMC function and bite force change with aging. It is important to evaluate how the activation of OMC and the function of other brain regions should be enhanced to prevent cognitive decline. We hypothesized that improving oral motor function can improve brain function. This study was conducted in the sitting position. The association between the left and right OMC function and bite force in the supine position in the same participants can be an interesting topic of future research. Using APR, it is possible to compare the resting brain activity status of OMC in the sitting and supine positions without using an oral motor task.

Oral movements should be assessed in the sitting position as it is advantageous to evaluate differences in one-sided biting habits in an environment similar to real-life behavior. In the current study, the participants had a higher *ΔOxyHb* in the ipsilateral OMC than in the contralateral side. There was no difference in APR and *ΔDeoxyHb* between the left and right sides during left-side bite, and the ipsilateral side increased more than the contralateral side in the right-side bite. The results could have been affected by the fact that 78.1% of participants had a right bite CSP. An fMRI study on tongue movement was reported by determining the participant’s CSP and extracting the left CSP group (Shinagawa, et al., 2004). Results showed that when participants moved their tongues after chewing gum, activation increased in the motor and sensory cortices ipsilateral to the CSP side. Data on the right side of the CSP group were not described.

In this study, the association between the CSP and the left and right OMCs was not analyzed. Although a sufficient number of participants are required, the association between CSP and the left and right OMCs is an interesting research topic.

### Potential for the zero-set vector-based fNIRS study

The use of APR values obtained *via* zero-set vector fNIRS is a novel method for evaluating differences in the resting state of the brain. Using APR, the resting state of oxygen metabolism can be simultaneously quantified from multiple sites with time resolution in the order of milliseconds.

fNIRS has been reported to be an effective method for neurorehabilitation compared to fMRI (Klein et al., 2022). However, when multiple sites are measured with a continuous wave multi-channel fNIRS system, the optical path lengths of each site are treated as identical even if they are different. Moreover, the concentration change of each hemoglobin has been qualitatively mapped.

The measurement of optical path length using time-resolved spectroscopy (TRS) or phase-resolved spectroscopy requires several minutes at rest. Hence, changes in the order of milliseconds or meters were not quantified in real time. In fact, the TRS takes approximately 5 min to measure the resting cerebral oxygen saturation at one location on the scalp. Using the OxyHb and DeoxyHb signals to calculate changes in tissue saturation (Robu et al., 2020) and to compare them to the time course of the APR may characterize the physiology of the APR and confirm that they are excellent markers. The physiological significance of APR values in the resting state *via* zero-set vector analysis should be further evaluated.

The time course of the APR can more accurately indicate bite task-related responses in OMC than *ΔOxyHb* or *ΔDexyHb.* Baseline normalization and motion correction as preprocesses for analysis may distort the phase of oxygen exchange (Kato, 2021). Hence, only data smoothing of *ΔOxyHb* and *ΔDexyHb* concentrations at 0.1-Hz low-pass filter was performed.

The phase *k* and norm *L* are obtained from the zero-set vector. Since the norm *L* reflects the signal intensity, the motion artifact may be more susceptible than the phase *k*. By contrast, phase *k* is less sensitive to trends in signal strength (Kato, 2021).

The technical challenges in fNIRS measurements are motion artifact and skin blood flow correction. Therefore, researchers have developed several artifact correction processes, such as wavelet-based motion correction (Perpetuini et al., 2021). The association between zero-set vectors and measurement noise should be further assessed.

### Study limitations

Scalp blood flow was not simultaneously measured. The extent to which APR and *L* obtained from the zero-set vectors that are affected by the use of preprocessing techniques must be determined.

If the population in Eq. 5 is greater, the APR can be calculated more accurately. Hence, the accuracy of the APR is dependent on the population of the zero-set vector group.

The APR was calculated as the population in all 140 trials. However, this study did not determine the minimum number of trials sufficient for analysis.

Determining the minimum number of trials required to unambiguously detect a task-dependent response by the APR could allow for an appropriate number of tasks in the experimental design. The APR may provide accurate quantitative values even with a small number of trials.

In this study, the duration of the resting period was only a few seconds, which was insufficient to assess each site at rest. A controlled study at rest for more than 1 min could be useful in the future.

This study was conducted using a pre-measured identification method (Murakoshi and Kato, 2006) of target cortical sites using the distance between reference points on the scalp from the participant’s MRI to place the measurement probes. Therefore, only one participant underwent MRI after the measurement to validate the OMC location. fNIRS is a measurement method, using a probe pair attached to the scalp. The brain region where the probe pair can detect the signal is limited to the nearest region of the probe pair. Therefore, it is necessary to detect location information with a higher accuracy in preparation for probe placement.

A recent study has assessed the optimization of fNIRS optodevice placement based on a transcranial brain atlas (Zhao et al., 2021). Pre-identifying transcranial brain regions that must be measured on MRI images from a population may be possible even with a portable type of fNIRS with a reduced number of channels, with good accuracy. In addition, it can help reduce the participants’ burden and research costs.

## Conclusion

Unlike conventional analysis methods, which are dependent on *ΔOxyHb* and *ΔDeoxyHb* signal intensities, APR can reflect the state of oxygen metabolism and can quantify and monitor changes in the resting state of the brain at each measurement site. In comparison to APR, ΔOxyHb and ΔDeoxyHb alone may overestimate the spatial extent of brain activity more broadly or the duration of brain activity longer. Using the zero-set vector-based fNIRS, APR can be a valid indicator to evaluate the relationship between OMC activity and oral motor function and bite force.

## Data Availability

The raw data supporting the conclusions of this article will be made available by the authors, without undue reservation.
